# Study of the Electronic Interaction between NiO and Short Polythiophene Chains towards Solar Photon Harvesting

**DOI:** 10.3390/ijms24119109

**Published:** 2023-05-23

**Authors:** Guillermo Carbajal-Franco, María Fernanda Márquez-Quintana, Hugo Rojas-Chávez, Alan Miralrio

**Affiliations:** 1 Tecnológico Nacional de México, Instituto Tecnológico de Toluca, Division of Graduate Studies and Research, Av. Tecnológico s.n., Metepec Estado de México 52149, Mexico; 2 Tecnológico Nacional de México, Instituto Tecnológico de Chilpancingo, Av. José Francisco Ruiz Massieu No. 5, Colonia Villa Moderna, Chilpancingo de los Bravo 39090, Mexico; 3 Tecnológico Nacional de México, Instituto Tecnológico de Tláhuac II, Camino Real 625, Col. Jardines del Llano, San Juan Ixtayopan, Alcaldía Tláhuac, Mexico City 13550, Mexico; 4 Tecnologico de Monterrey, Escuela de Ingeniería y Ciencias, Ave. Eugenio Garza Sada 2501, Monterrey 64849, Mexico

**Keywords:** polythiophene, nickel oxide, photon harvesting, solar cell, nickel monoxide

## Abstract

The sustainable production of energy is a field of interest to which a new requirement is now imposed: the need to be respectful of the environment. New materials and techniques are being developed, but environmental concerns impose the necessity of keeping research active towards the development of green energy. For this reason, we present the study of short polythiophene (PTh) chains (three and five monomers) and their interaction with nickel oxide, looking for properties related to solar photon harvesting in order to produce electricity. The models of the molecules were developed, and the calculations were performed with an M11-L meta-GGA functional, specially developed for electronic structure calculations. The theoretical explorations demonstrated that the geometry of the PTh molecules suffer little distortion when interacting with the NiO molecule. The calculated value of *E*_g_ lies between 2.500 and 0.412 eV for a three-ring PTh chain and between 1.944 and 0.556 eV for a five-ring PTh chain. The chemical parameters indicated that, depending on the geometry of the system, the chemical potential varies from 81.27 to 102.38 kcal/mol and the highest amount of electronic charge varies from −2.94 to 21.56 a.u. for three-monomer systems. For five-monomer systems, the values lie within similar ranges as those of the three-monomer systems. The Partial Density of States (PDOS) showed that the valence and conduction electronic bands were composed of states in the NiO and PTh rings, except for a system where there was a non-bonding interaction.

## 1. Introduction

Solar energy harvesting is a widely studied topic that requires continuous upgrades due to the vast research opportunities derived from the multidisciplinary fields that have demonstrated a direct impact on it. Far from the well-known fields of chemistry and physics, it is now considered an intrinsic part of materials science and new tools have been incorporated into the research, for instance, AI and computational modeling from computer science. These tools have demonstrated a great step forward because they help to develop and theoretically test materials. Furthermore, methods and complete systems, before investing any resources into their fabrication, have also been studied. This has resulted in great savings, not only in terms of monetary resources, but also in terms of large amounts of wasted time because no useful data can be studied from most failed experiments.

It is also well-known that polymers are materials with a wide range of applications, because the semiconducting properties that some of them have demonstrated are—mainly—due to the alternation of single (σ) and double (π) C-C bonds along the polymer backbone chain [1]. The most studied semiconducting polymers are polyfuran (PF), polyaniline (PANI), polyacetylene (PA), polypyrrole (PPy), poly(-phenylenevinylene) (PPV), polythiophene (PTh), and poly(para-phenylene) (PPP) [2]. From the previous list of conducting polymers, PTh is a material with interesting properties that have surpassed the line of promising properties and led to successful applications. For instance, it has been used for biomedical applications [3], as neural probes [4], tissue engineering, supercapacitors, photonic applications, and biosensors [3], among others. It is worth highlighting that PTh is widely used. Consequently, the list of applications keeps growing due to the possibility of doping it with different materials, which can enhance the properties of the sole polymer [5,6,7,8,9,10,11].

In the field of solar energy harvesting, there have been many attempts to fabricate solar cell-devices by enhancing the properties of conjugated polymers [12,13]. In the case of PTh, this is achieved by adding silver to get the metallic atoms into the PTh interlayers [14], mixing TiN/TiO_2_-PTh composites for use as photo-anodes in solar cells and water splitting devices [15], adding or mixing with different oxides, such as perovskites [16], and coating TiO_2_ nanoparticles [17], changing the electronic properties by including other compounds or elements [18,19], and making blends of different polymers and other organic compounds, such as fullerenes [20,21,22,23,24,25]. In this context, nickel oxide is a material that has been largely studied for its applications, such as electrodes for supercapacitors [26] and many others [27]. The composite Ni–PTh has been studied to tune the electronic properties of the mixture by means of its production [8]. Specifically, the NiO–PTh composite has been studied as an anode for a biofuel cell [28], but the specific application of the NiO–PTh composite has not been studied as material for solar cells, neither as an electrode nor as an active material. Consequently, the purpose of this research was to study the variations in the electronic properties of polythiophene short chains when interacting with NiO molecules. It is worth noting that the literature does not mention the number of monomers on the presented polymeric chains. Thus, in this work, the short chain polymers can also be considered as oligomers.

This work demonstrates that electronic properties are developed due to the interaction between the short chains of polythiophene molecules and nickel oxide, which are within the range of values that are appropriate for use in solar harvesting, such as solar cells and photocatalytic applications.

## 2. Results and Discussion

As has been previously said, one of the most useful advantages of modeling is the possibility of calculating many different parameters and correlating them in order to conclude whether a material is viable for a specific application. The interaction between NiO and the thiophene monomer has been documented elsewhere [29]. In the present research, we investigated several parameters of two basic models of the short chains of PTh or oligothiophenes, one set of three monomers and a second set consisting of five monomers. First, we found the parameters of the sole oligomer and then the interaction between the oligothiophene and nickel oxide. In order to cover all the possible interactions between the molecules, many different positions of the NiO molecule in relation to the polymer were attempted, all of which always resulted in one of four final geometries that we propose in this work.

### 2.1. Geometry Optimization

Different configurations were studied as starting systems, in which the position of the NiO molecule relative to the PTh molecule was changed, taking into account the symmetry of the central monomer of the polymer and the oxide molecule. As mentioned before, all the starting geometries always resulted in one of the four final stable geometries that are presented in the following sections.

Table 1 summarizes the total energies of the systems after optimizing their geometries at 0 K, the free energy at 298.15 K, and the total free energy corrected for a temperature of 298.15 K.

#### 2.1.1. Geometry of the 3PTh Systems

**Figure 1:**
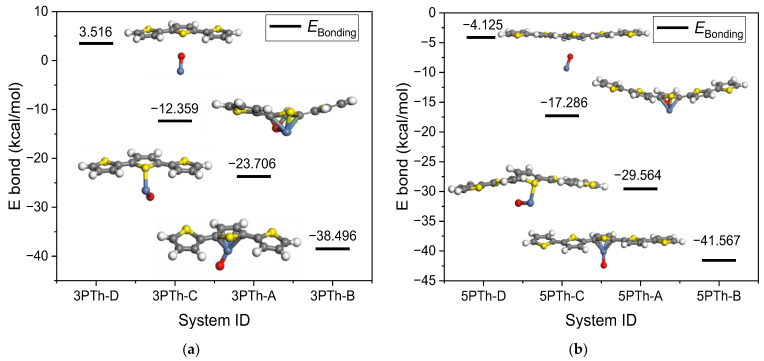
Optimized geometry and bonding energy of the systems composed by a short chain of polythiophene and an NiO molecule: (**a**) 3PTh-NiO bonding energy; (**b**) 5PTh-NiO bonding energy.

The first system was composed of three monomers of thiophene (3PTh), and the resulting molecule was slightly bent along the backbone of the polymer. In the four following systems, the NiO molecule was placed on the central monomer of the polymer chain with the Ni atom near the S atom of the central thiophen, changing the position of the O atom with respect to the C atoms. The second system (3PTh-A) was composed of a 3PTh molecule which interacted with an NiO molecule linked to the 3PTh, forming an Ni–S bond and causing the O to move far from the polymer. In the third system (3PTh-B), the Ni atom bonded to the four C atoms of the central monomer of the 3PTh molecule. In the fourth system (3PTh-C), the final geometry was similar to that of the 3PTh-A system, but in this case, the O atom bonded to the two C atoms that were in the back of the central monomer of the molecule. Moreover, the Ni atoms also bonded to the two C atoms at the sides of the S atom, which also bonded to the Ni atom. Finally, in the fifth system (3PTh-D), there was no bond between the 3PTh molecule and NiO. It is worth noting that the non-bonding interaction of the molecules always occurred when the O atom was pointing to the polymer, whereas in all the other cases, the Ni was closer to the polymer or was at least at the same distance as the O atom. All the configurations are presented in Figure 1a. It can be seen that the interaction between molecules only introduces a slight bending of the polymer chains or a little twist along the length of the polymer, as both the changes were not large enough to modify the 1D nature of the short polymer chain.

#### 2.1.2. Geometry of the 5PTh Systems

In the case of the oligothiophene with five monomers, the final configuration remained unchanged compared to the systems with three monomers. The 5PTh system was composed of a sole polymer, which, in this case, did not bend but slightly twisted around the backbone of the polymer. The 5PTh-A system also presents the Ni atom bonded to the S atom of the central monomer. In this sense, the 5PTh-B system corresponds to the Ni bonded to the four C atoms of the central monomer. The 5PTh-C system also presents the NiO fully bonded to the central monomer of the polymer chain, as in the 3PTh-D system, and the 5PTh-D system also corresponds to a non-interacting geometry of the molecules. All the configurations are shown in Figure 1b. The changes produced to the molecules due to the NiO–PTh interaction are similar to those observed in the 5PTh systems, but as they were larger, bending of the 5PTh-A and 5PTh-C systems was more obvious. This fact makes it evident that the type of bonding of the molecules impacts the geometry of the polymer in different ways. In the 5PTh-A and 5PTh-B systems, where only the Ni atom directly bonded to the polymer chain, the change in the geometry corresponds to a twist in the opposite direction along the long axis of the polymer. In the 5PTh-C system, where the Ni atom bonded to the S atom and the O atom bonded to the four C atoms of the monomer, the polymer bent in the direction opposite to that of the NiO molecule.

### 2.2. Molecular Bonding Energy

#### 2.2.1. Molecular Bonding Energy of the 3PTh Systems

The bonding energy (*E*_Bonding_) of the 3PTh systems was calculated and is presented in Figure 1a. It should be noted that it is organized from lower to maximum bonding energy.

According to Figure 1a, the most probable energetically favorable system for the bonding between 3PTh and NiO is the 3PTh-B system, in which, based on the bonding energy, the Ni atom chemically bonds to the four carbon atoms of the central monomer with an energy of −38.496 kcal/mol. The 3PTh-B system presents a bonding energy that corresponds to the formation of a chemical bond with a value of −23.706 kcal/mol. This contrasts with the energies presented by the other two systems, which present characteristics of physisorption processes with values of −12.359 and 3.516 kcal/mol for the 3PTh-C and 3PTh-D systems, respectively. It is important to notice that, in the latter case, the positive value of the energy means that a slight amount of energy is needed for the polymer to interact with the NiO molecule in a non-bonding way, with the oxygen atom pointing towards the organic molecule. From the calculated energies, it is clear that the spontaneous chemisorption of the NiO on the 3PTh molecule occurs only when the Ni atom is the only one that bonds to the polymer.

#### 2.2.2. Molecular Bonding Energy of the 5PTh Systems

The bonding energy between the NiO and polymer molecules composed of five monomers is shown in Figure 1b. It is evident from the results that the behavior of the energy of the 5PTh systems follows the same trend as the polythiophene systems with three monomers. The main differences are the higher bonding energies for similar systems and the fact that the value of −4.125 kcal/mol for the 5PTh-D system makes the spontaneous occurrence of the non-bonding molecular interaction possible, unlike the case of the 3PTh-D system, which requires a slight amount of energy to occur.

### 2.3. Molecular Orbitals and Band Gap Energies

**Figure 2:**
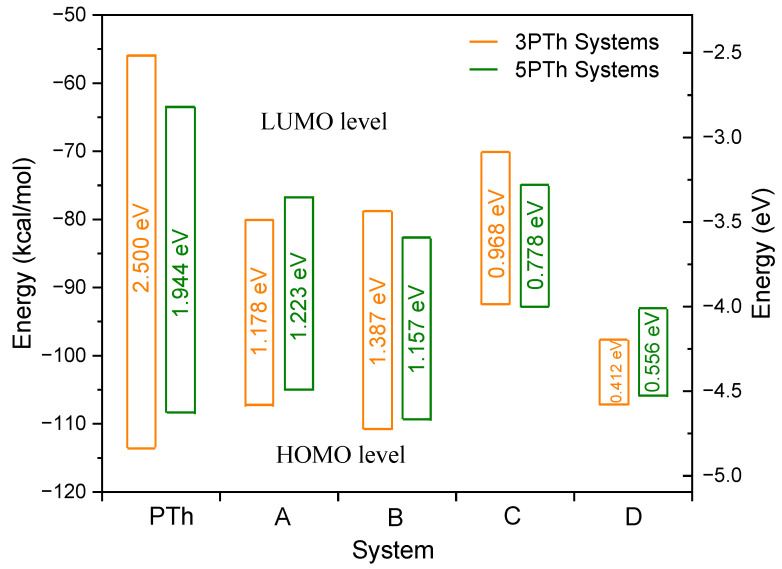
Energy values of the HOMO–LUMO orbitals and the energy band gap (*E*_g_) between the orbitals of the 3PTh and 5PTh systems. The energy gap values are presented in eV. The orange bars correspond to the 3PTh system and the green bars correspond to the 5PTh system.

Figure 2 shows the value of the HOMO–LUMO orbitals and the energy band gap (*E*_g_) between the orbitals of the 3PTh and 5PTh systems.

It is noticeable that, as for the *E*_Bonding_, the orbitals of the systems behave in similar ways according to the geometry of the molecules. The systems with the highest *E*_g_ correspond to the sole PTh molecule with values of 1.944 and 2.500 eV for three and five monomers, respectively. When the NiO molecule was introduced to the systems, the value of *E*_g_ reduced down to 52.88% and 37.09% for the 3PTh-A and 5PTh-A systems, respectively. The values for the B systems remained almost the same, with the 3PTh-B system showing a slight reduction in its *E*_g_ value from 1.223 down to 1.157 eV. The 5PTh-B system increased its *E*_g_ value from 1.178 to 1.387 eV. The systems with the whole NiO molecule attached to the central PTh monomer exhibited considerable reductions of 0.778 and 0.968 eV for the 3PTh-C and 5PTh-C systems, respectively. The systems with no apparent intermolecular interaction presented the smallest *E*_g_ values of 0.556 and 0.412 eV for the three- and five-monomer systems, respectively.

#### 2.3.1. HOMO–LUMO Orbitals of the 3PTh Systems

**Figure 3:**
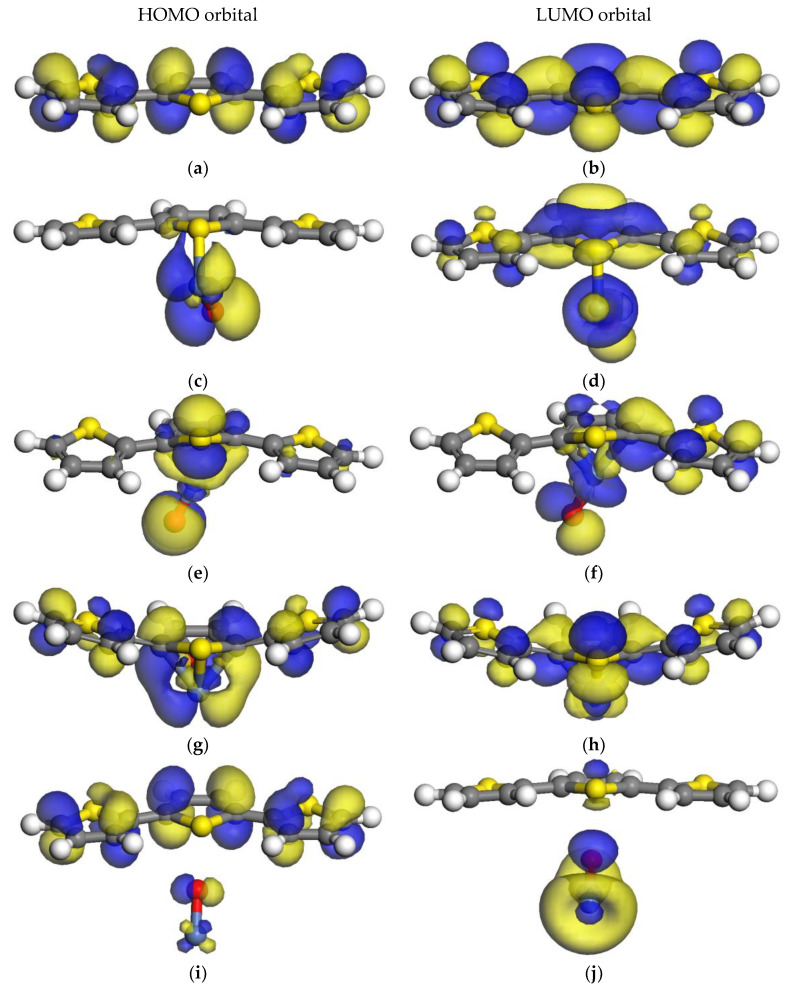
HOMO and LUMO orbitals of three-monomer chain systems: (**a**,**b**) 3PTh HOMO–LUMO orbitals; (**c**,**d**) 3PTh-A HOMO–LUMO orbitals; (**e**,**f**) 3PTh-B HOMO–LUMO orbitals; (**g**,**h**) 3PTh-C HOMO-LUMO orbitals; and (**i**,**j**) 3PTh-D HOMO–LUMO orbitals. Note that the gray atoms are carbon atoms, the blue atoms are nickel atoms, the yellow atoms are sulfur atoms, and the white atoms are hydrogen atoms.

For the single polymer molecule shown in Figure 3a,b, the HOMO orbital is perpendicular to the long axis of the monomer chain, whereas the LUMO orbital is parallel to the same axis, opening the possibility of photo-generated electrons moving along the polymer.

In the 3PTh-A system shown in Figure 3c,d, the HOMO level was located around the NiO molecule, but the LUMO level was located in the NiO molecule and the central monomer of the chain. Hence, it is possible for the exited electrons, mainly from the NiO, to travel all along the material. In contrast to the 3PTh-B system (Figure 3e,f), where the electrons on the HOMO level are distributed between the central monomer and the NiO molecule, the LUMO level was also present in the NiO and in one side of the polymer chain. Figure 3g,h shows the system with the most uniform presence of the HOMO–LUMO orbitals. In this system (3PTh-C), the electrons of the valence band are well distributed all along the system, and when these electrons get excited, they can jump to the LUMO level that is also present along the whole system. As for the 3PTh-D system shown in Figure 3i,j, the non-bonding interaction between the molecules (where shortest distance between the molecules was 2.584 Å, formed by the O and the S atoms) presents major contributing HOMO orbitals from the polymer molecule, and the relevant LUMO orbital corresponds to the nickel oxide.

According to the spatial distribution of the HOMO–LUMO orbitals and the *E*_g_ values between them, the system with the best distribution of the orbitals is the 3PTh system. However, it is the system with the largest *E*_g_. The system with the lowest *E*_g_ value was the 3PTh-D system. However, the electrons will get trapped in the NiO molecule because most of the LUMO orbital is contributed by this molecule (Figure 3j). The 3PTh-C system is the one that presents the lowest *E*_g_ and the best HOMO–LUMO distribution all along the molecules.

#### 2.3.2. HOMO–LUMO Orbitals of the 5PTh Systems

**Figure 4:**
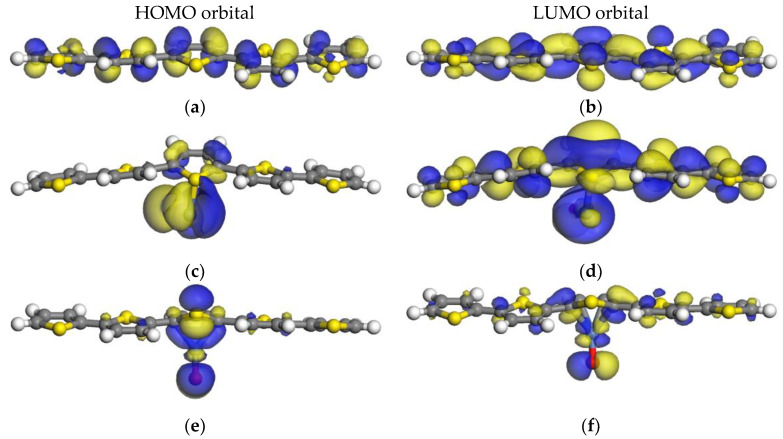

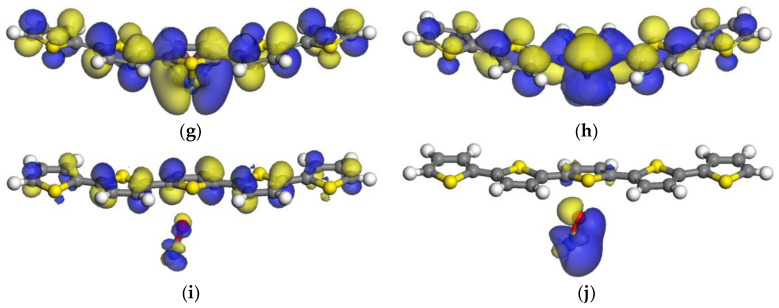
HOMO and LUMO orbitals of five-monomer chain systems: (**a**,**b**) 5PTh HOMO–LUMO orbitals; (**c**,**d**) 5PTh-A HOMO–LUMO orbitals; (**e**,**f**) 5PTh-B HOMO–LUMO orbitals; (**g**,**h**) 5PTh-C HOMO–LUMO orbitals; and (**i**,**j**) 5PTh-D HOMO–LUMO orbitals. Note that the gray atoms are carbon atoms, the blue atoms are nickel atoms, the yellow atoms are sulfur atoms, and the white atoms are hydrogen atoms.

In the systems with polymer chains formed by five monomer units (Figure 4), the shape of the HOMO–LUMO orbitals closely follow the distribution presented by the systems constituted by three monomers. The differences lie in the extension of the LUMO orbitals. Even though the energies of the orbitals are similar in the three- and five-monomer systems, they cover a larger extension of the five polymer rings, except for the 5PTh-D system where the LUMO orbital on the polymer is around the C atoms at both sides of the sulfur on the central monomer.

For these larger polymers, the 5PTh-C system remains the most promising for solar cell applications. The 5PTh-A system could also be considered since, in this case, the electrons from the NiO can be distributed all along the polymer chain because the LUMO orbitals extend to the monomers at both ends of the chain.

### 2.4. Global Molecular Reactivity

The chemical parameters presented in this section were calculated in accordance with the frontier-electron theory of chemical reactivity [30], which is described in Section 3.

#### 2.4.1. Molecules Constituted by Three Monomers

Table 2 shows the chemical parameters calculated for the systems with a three-monomer chain. Additionally, Table 2 also presents the bonding energy, HOMO–LUMO energy and *E*_g_ values.

The chemical potential value *μ* indicates that the 3PTh-D system is more prone to donating electrons, whereas the 3PTh-C system is the less likely to donate them, indicating that it is more susceptible to accepting negatively charged carriers. In terms of the hardness (*η*) and softness (*S*), the system that is less likely to suffer changes, such as the photo-generation of electric charge, is the 3PTh system. In contrast, the system that is most susceptible to changes is the 3PTh-D system, where the NiO molecule is not bonded to the polymer. The bonding of NiO to the polymer in this system is a possible change that could occur, as reflected by its low hardness value. The difference presented in electrophilicity (*ω*) indicates that the sole polymer will not be able to receive the photo-generated electrons (*ω* = 124.66), but the addition of an NiO molecule to the surroundings, even with no bonding, greatly increases the avidity of the system for electrons (*ω* = 1103.58102). The values of ∆*N*_max_ indicate that the 3PTh-D system can accommodate a larger change to the electrons than the other four systems.

#### 2.4.2. Molecules Constituted by Five Monomers

The values of the global molecular reactivity parameters for the five-monomer systems are shown in Table 3.

In the case of the systems with five monomers in the polymer chain, the systems A and C suffered the largest changes in chemical potential when increasing the number of thiophene rings from three to five. In this case, the former decreased by 9.78 kcal/mol and the later increased by 9.58 kcal/mol, which means that the 5PTh-A system is less prone to donating electrons than the same system with three monomers, and the 5PTh-C system is more susceptible to electron donation. This can be corroborated with the changes in electrophilicity and ∆*N*_max_, whose values indicate that the 5PTh-A system is more prone to receiving electrons than the 3PTh-A system, whereas the 5PTh-C system can accommodate a small number of electrons. The rest of the systems suffered less significant changes in the values of the chemical parameters, except for the 5PTh-D system, whose *μ* and *η* values suffered small changes, but the electron-related parameters *ω* and ∆*N*_max_ were drastically reduced.

### 2.5. Molecular Electrostatic Potential (MEP)

**Figure 5:**
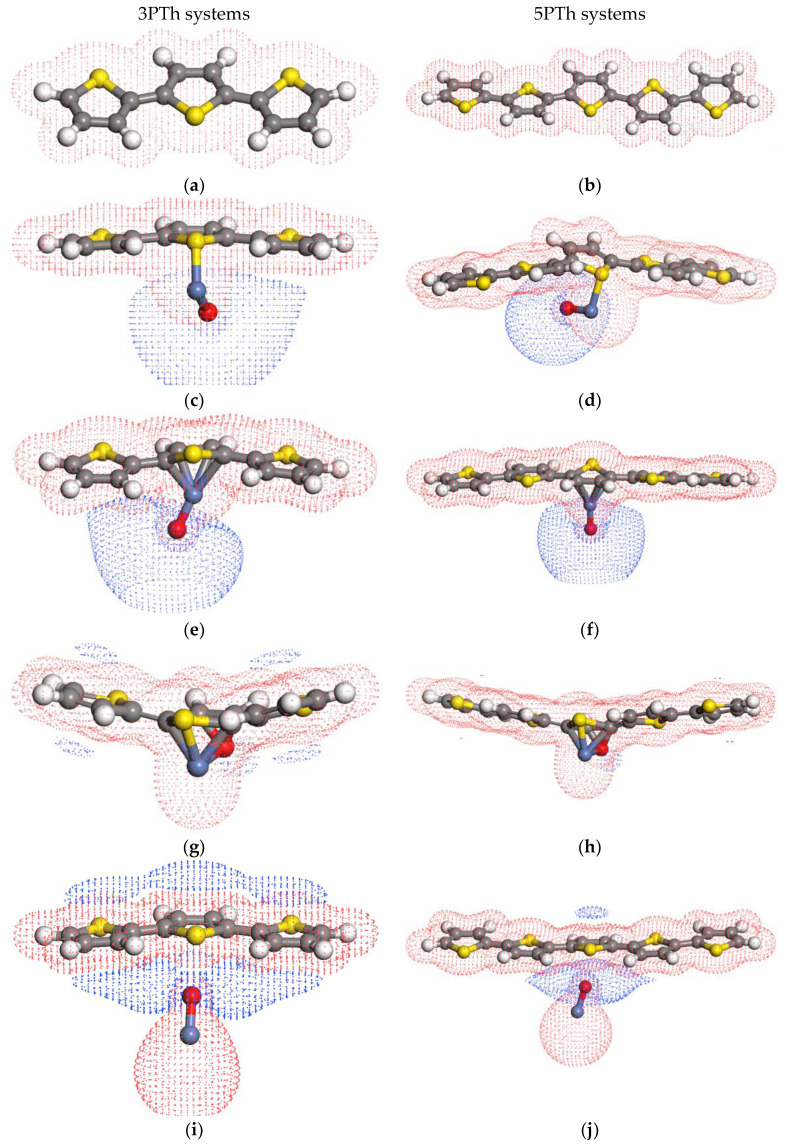
Molecular electrostatic potential (MEP) systems composed of a short chain of polythiophene and an NiO molecule: (**a**,**b**) sole polymer molecules; (**c**,**d**) A systems with three and five monomers; (**e**,**f**) B systems with three and five monomers; (**g**,**h**) C systems with three and five monomers; and (**i**,**j**) D systems with three and five monomers. Note that the gray atoms are carbon atoms, the blue atom is a nickel atom, the yellow atoms are sulfur atoms, and the white atoms are hydrogen atoms. Isosurfaces were plotted at 0.035 a.u. It is worth noting that the red dots represent a positive electrostatic field, whereas the blue dots correspond to a negative electrostatic field.

Figure 5 shows the molecular electrostatic potential (MEP) of the three- and five-monomer systems. In the systems composed of a sole polymer, the MEP was positive all along the molecule, indicating its avidity for electrons. For the A and B systems (Figure 5c,f), the MEP was positive all around the polymer and the nickel, but it was negative around the O atom, clearly distinguishing two different electrostatic field zones in the system. This electric field distribution will improve the separation of the electric carriers [31,32,33] once they are photo-generated by the incidence of electromagnetic radiation, according to the calculated wavelengths for the system.

In the case of the C systems depicted in Figure 5g,h, the size of the zone with a negative MEP value is much smaller than the zone with a positive MEP value. Therefore, it is expected that a reduced effect of charge separation in this system would be found, no matter the number of monomers in the polymer chain. For the D systems (Figure 5i,j), the O atom induces a considerable zone with a negative MEP above and below the polymer chain that almost covers its entire length. However, this is not the case for the D system with five monomers, where the negative MEP zones are barely below the equivalent of two monomers, and the zone above the chains is no larger than one monomer. This makes the system of five TH monomers less likely to effectively separate the photo-generated electric carriers.

### 2.6. Partial Density of States (PDOS)

**Figure 6:**
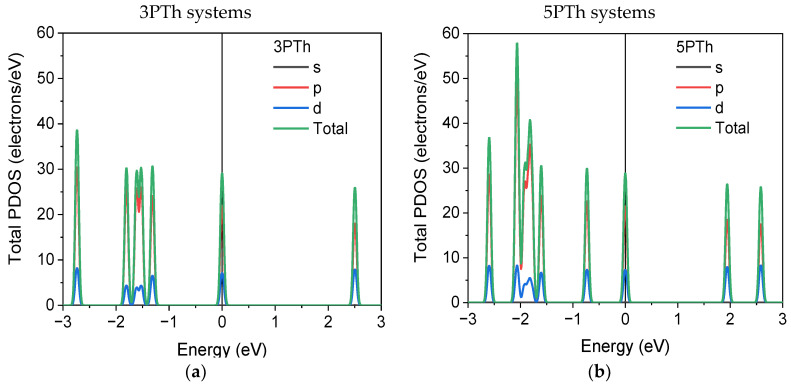

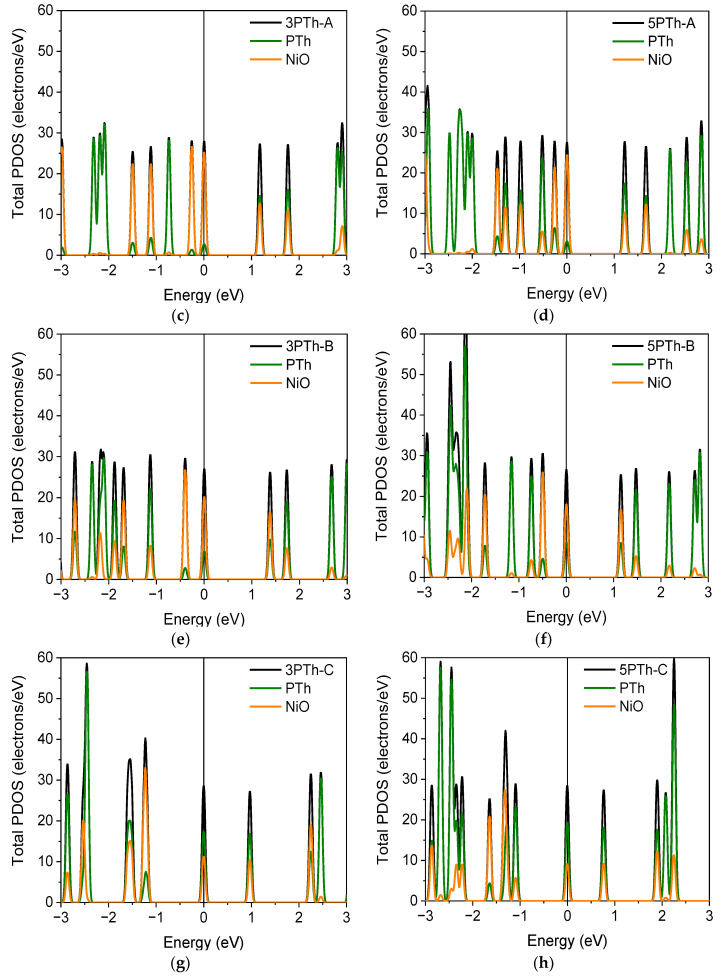

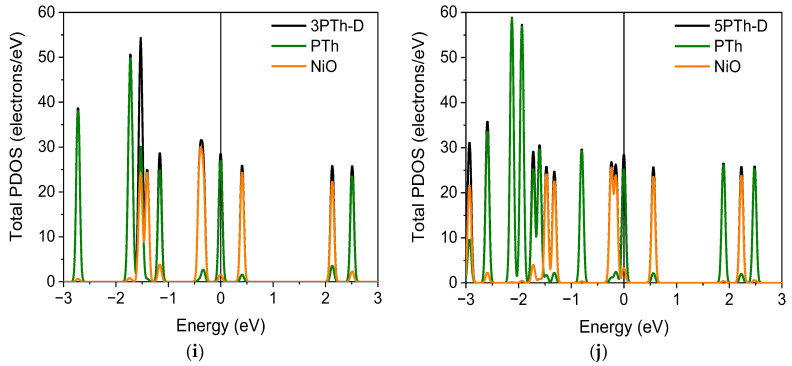
Partial Density of States (PDOS) and total DOS plots of the systems: (**a**,**b**) partial DOS of the sole polymer molecules. For the rest of the plots, the green lines correspond to the PTh-chain, the orange lines represent contributions from the NiO, and the black lines correspond to the DOS of the whole system. (**c**,**d**) A systems with three and five monomers; (**e**,**f**) B systems with three and five monomers; (**g**,**h**) C systems with three and five monomers; and (**i**,**j**) D systems with three and five monomers.

The results of the partial density of states (PDOS) of the 10 polymer–NiO systems are shown in Figure 6. In Figure 6a,b, the PDOS plots of the systems without NiO show that the conduction band (CB) and the valence band (VB), corresponding to the HOMO and LUMO orbitals, respectively, are composed mostly of *p* orbitals, whereas the *d* orbitals contribute to only approximately one third of the levels. In Figure 6c,d, the DOS of the A systems are shown; in this and in the following cases, the traces correspond to the total DOS of the whole system (black line), the DOS of the polymer chain (green), and the DOS of the NiO molecule (orange). In both the A systems, the VB is mainly composed of states on the NiO molecule. The differences between the systems with three and five monomers lies in the composition of the CB states. In the 3PTh-A system, the CB is composed of almost an equal number of states in the polymer and the oxide molecule, whereas the CB in the 5PTh-A system consists of 65.52% more states from the polymer than states corresponding to the NiO molecule.

The conduction and valence bands of the B systems with three and five monomers behave almost similarly; the bands are mostly composed of states in the NiO molecule, with the contribution of the polymer chain being less than one half of the total states of the system for both bands. The DOS composition of VB and CB for the C systems is completely different in comparison to the B systems. In these cases, the major contribution to the bands comes from the polymer molecule. Finally, in the D systems, as suggested by the HOMO–LUMO results (Figure 4i,j), the VB is basically composed of electronic states in the polymer. Conversely, the CB is almost completely composed of states in the NiO molecule.

## 3. Materials and Methods

### Computational Details

All the simulations were processed by DMol^3^ [34] as coded in Dassault Systèmes Biovia Materials Studio suite, Vélizy-Villacoublay, France, with the meta-GGA functional M11-L [35]. For the convergence tolerance of geometry optimization, the energy was set to 2 × 10^−5^ Ha, and a maximum force of 4 × 10^−3^ Ha/Å and a maximum displacement of 5 × 10^−3^ Å was allowed. The SCF tolerance was set to 10^−5^ using hexadecapole multipolar expansion. For the PDOS plots, a smearing of 0.03 eV was used.

All the energies used for the calculations were corrected with the free energy at 295.15 K (*E*_Corr_) by adding the free energy at 298.15 K *G*_298.15_ to the total free energy at 0 K *E*_0_. The equation used to calculate the bonding energy was:

(1) $$E_{\mathrm{Bonding}}=E_{\mathrm{System}}-\left(E_{\mathrm{Polymer}}+E_{\mathrm{NiO}}\right)$$

where *E*_Bonding_ is the energy required for the bonding interaction between molecules, *E*_System_ is the energy of the whole system with the polymer chain and the nickel oxide molecule, *E*_Polymer_ is the energy of the sole polymer of three or five monomers, depending on the system, and *E*_NiO_ is the energy of the sole NiO molecule. The geometries of all the systems and molecules were optimized.

For the global molecular chemical parameters, the equations used were:

(2) $$\mu =-\left(\frac{E_{\mathrm{LUMO}}+E_{\mathrm{HOMO}}}{2}\right)$$

(3) $$\eta =\frac{E_{\mathrm{LUMO}}-E_{\mathrm{HOMO}}}{2}$$

(4) $$S=\frac{1}{2\eta }$$

(5) $$\omega =\frac{\mu^{2}}{2\eta }$$

(6) $$\Delta N_{\max}=-\frac{\mu }{\eta }$$

where *E*_HOMO_ and *E*_LUMO_ are the energies of the HOMO and LUMO orbitals, respectively, *μ* is the chemical potential, *η* is the hardness, *S* is the softness, *ω* is the electrophilicity, and ∆*N*_max_ is the highest amount of electronic charge that the system can receive, calculated following the frontier-electron theory of chemical reactivity [30], where the ionization potential and the electron affinity were approximated using the HOMO and LUMO energies, respectively [36].

## 4. Conclusions

The modeling of many different geometries for studying the interaction between an NiO molecule and the short chain of polythiophene molecules, with three and five monomers per polymer chain, resulted in ten different systems. These systems were modeled and optimized with the aim of studying the convenience of their properties for use as materials for solar photon harvesting. The results showed that the most stable systems were the ones in which the nickel atom of NiO bonded to the C atoms of one ring in the polymer (B systems). There were also configurations (D systems) that were stable without bonding between the molecules, which happened when the oxygen of the oxide faced the monomer. In terms of the band gap energy, the shortest values of 0.412 (three monomers) and 0.556 eV (five monomers) for the D systems indicated materials that can absorb the radiation of large wavelengths, whereas the band gap of 1.387 eV for the 3PTh-B system indicated that it can absorb radiation near the visible region and of the shortest wavelengths. The chemical potential showed that the ability to donate electrons is dependent on the number of monomers in the polymer only when the nickel atom of NiO bonds to one sulfur atom of the polymer chain, without affecting the electrophilicity in the case of the C system, where the chemical potential increased with the number of monomers in the polymer, but the electrophilicity remained almost the same. The analysis of the MEP showed that there were no differences in the potential of the sole polymers. However, in the case of the NiO that was not bonded to the polymer, or when the NiO was bonded only by the Ni atom, there were highly differentiated zones of electrostatic potential. The DOS analysis showed that the VB and CB were mostly composed of states from the two molecules composing the systems, except for the D systems. In the D systems, the VB was mostly composed of states on the polymer and the CB was composed of states in the non-bonded NiO molecule. This situation may not be favorable because the electrons would accumulate in the NiO, where they would have a short mean free path.

## Figures and Tables

**Table 1:** Total energies and free energies of three and five PTh ring systems. Total energy at 0 K (*E*_0_), free energy at 298.15 K (*G*_298.15_), and total free energy corrected for 298.15 K (*E*_298.15_). All the energies are presented in kcal/mol.

3PTh	*E*_0_	*G*_298.15_	*E*_298.15_	5PTh	*E*_0_	*G*_298.15_	*E*_298.15_
A	−2,033,052	77.165	−2,032,975	A	−2,725,485	128.79	−2,725,356
B	−2,033,066	77.110	−2,032,989	B	−2,725,499	130.463	−2,725,369
C	−2,033,038	74.584	−2,032,963	C	−2,725,473	129.148	−2,725,344
D	−2,033,024	76.239	−2,032,948	D	−2,725,459	128.037	−2,725,331

**Table 2:** Three PTh rings. The energy of adsorption (*E*_Bonfing_), HOMO and LUMO energy levels (*E*_HOMO_ and *E*_LUMO_, respectively), forbidden electronic band gap (*E*_g_), chemical potential (*μ*), hardness (*η*), softness (*S*), electrophilicity (*ω*), and the highest amount of electronic charge (∆*N*_max_) were calculated following the frontier-electron theory of chemical reactivity [30].

ID	*E*_Bonfing_	*E*_HOMO_	*E*_LUMO_	*E*_g_	*μ*	*η*	*S* (×10^−2^)	*ω*	∆*N*_max_
3PTh		−113.60	−55.95	−57.65 (2.5)	84.77	28.82	1.73	124.66	−2.94
A	−23.71	−107.22	−80.05	−27.17 (1.18)	93.64	13.58	3.68	322.76	−6.89
B	−38.50	−110.77	−78.78	−31.98 (1.39)	94.77	15.99	3.13	280.86	−5.93
C	−12.36	−92.43	−70.11	−22.32 (0.97)	81.27	11.16	4.48	295.91	−7.28
D	3.52	−107.13	−97.63	−9.50 (0.41)	102.38	4.75	10.53	1103.58	−21.56

All the quantities are presented in kcal/mol, with the exception of ∆*N*_max_, which is expressed in a.u. *E*_g_ is given, in parentheses, in eV.

**Table 3:** Five PTh rings. The energy of adsorption (*E*_Bonfding_), HOMO and LUMO energy levels (*E*_HOMO_ and *E*_LUMO_, respectively), forbidden electronic band gap (*E*_g_), chemical potential (*μ*), hardness (*η*), softness (*S*), electrophilicity (*ω*), and the highest amount of electronic charge (∆*N*_max_) were calculated following the frontier-electron theory of chemical reactivity [30].

ID	*E*_Bonding_	*E*_HOMO_	*E*_LUMO_	*E*_g_	*μ*	*η*	*S* (×10^−2^)	*ω*	∆*N*_max_
5PTh		−108.32	−63.48	−44.84 (1.94)	85.90	22.42	2.23	164.58	−3.83
A	−17.29	−92.83	−74.90	−17.93 (0.78)	83.86	8.97	5.58	392.17	−9.35
B	−41.57	−109.34	−82.65	−26.69 (1.16)	96.00	13.35	3.75	345.26	−7.19
C	−29.56	−104.95	−76.76	−28.20 (1.22)	90.85	14.10	3.55	292.75	−6.44
D	−4.13	−105.87	−93.04	−12.83 (0.56)	99.45	6.42	7.79	770.86	−15.50

All the quantities are presented in kcal/mol, with the exception of ∆*N*_max_, which is expressed in a.u. *E*_g_ is given, in parenthesis, in eV.

## Data Availability

Not applicable.
